# Rapid Fc-mediated surface modification of liposomes with antibodies for selective targeting of pancreatic tumors^☆^

**DOI:** 10.1016/j.jconrel.2026.115319

**Published:** 2026-08-31

**Authors:** Ibrahim Knani, Yasmin Habib, Galoz Kaneti, Orly Shayderman, Mohammed Alyan, Tasneem Abu-Raiya, Jeny Shklover, Avi Schroeder

**Affiliations:** a The Louis Family Laboratory for Targeted Drug Delivery and Personalized Medicine Technologies, Department of Chemical Engineering, Technion - Israel Institute of Technology, Haifa 3200003, Israel; b The Institute of Applied Research, The Galilee Society, Shefa-Amr, Israel; c Medical Research Institute, The Holy Family Hospital, Nazareth 1641100, Israel^1^; d The Norman Seiden Multidisciplinary Program for Nanoscience and Nanotechnology, Technion - Israel Institute of Technology, Haifa 32000, Israel

**Keywords:** Nanotechnology, Liposome, Targeting, Antibody, Cancer, PDAC

## Abstract

This study introduces a versatile platform for targeted drug delivery that addresses the complexities of conventional immunoliposome preparation. Traditional methods for conjugating a monoclonal antibody (mAb) to the surface of liposomes require target-specific chemical modification for each antibody, frequently resulting in suboptimal ligand orientation, steric hindrance of the antigen-binding sites, and a profound lack of modularity across different targeting systems. We developed Universal Particles (UPs), which are liposomal nanocarriers displaying high-affinity secondary antibodies on their surface. This design enables rapid, non-covalent functionalization by instantly binding to the constant (Fc) region of any whole-molecule primary targeting antibody without the need for chemical modification, thereby promoting a highly accessible, outward-facing display of the antigen-binding domains.

In vitro experiments confirmed that UPs successfully functionalized with primary antibodies, leading to specific molecular recognition and enhancing cellular uptake of anti-Muc1/Muc4 UPs by Panc02 pancreatic cancer cells. Analytical flow cytometry confirmed the high expression of these target antigens in the Panc02 line, with 99.5% of cells expressing Muc1 and 99.9% expressing Muc4. In vivo biodistribution studies validated the platform’s versatility: αCD31 UPs showed increased accumulation in the lungs, while dual anti-Muc1/Muc4 UPs demonstrated significantly enhanced tumor accumulation in a murine pancreatic tumor model compared with untargeted controls.

The UP platform offers a simple approach for antibody-based liposomal targeting, providing a foundation for targeted delivery systems aimed at enhancing therapeutic selectivity and reducing off-target effects in preclinical models.

## 1 Introduction

Cancer remains a leading cause of mortality worldwide, with poor drug solubility, short circulation half-lives, and off-target side effects that damage healthy tissues and narrow the therapeutic window [1–8]. To overcome these challenges, nanomedicine-particularly liposomal drug delivery - has emerged as a clinically translated platform for optimizing drug biodistribution and extending in vivo circulation [9–15].

While first-generation PEGylated liposomal formulations achieve passive tumor accumulation via the enhanced permeability and retention (EPR) effect, active targeting systems decorated with cell-specific ligands are considered highly effective because of the ligands’ strong affinity for specific target proteins and stability in systemic circulation [16–18]. Indeed, antibody-drug conjugates (ADCs) have received regulatory FDA approval [19]. However, the clinical use of ADCs is limited by solubility and degradation issues in the blood and the challenges associated with site-specific antibody conjugation [20].

To address these challenges, immunoliposomes (antibody-conjugated liposomes) are considered a viable active targeting drug delivery system [21]. However, traditional approaches to immunoliposome preparation primarily rely on the direct, covalent linkage of antibodies to liposomal phospholipids [26]. This methodology presents a significant performance bottleneck: chemical conjugation typically occurs in a random fashion, leading to suboptimal antibody orientation in which the critical antigen-binding (Fab) domains may be sterically blocked or forced to face inward toward the lipid bilayer [13,22]. Such random packing drastically reduces the density of functional binding sites, compromising the particle’s overall avidity and target-binding performance [23]. Furthermore, conventional covalent conjugation protocols must be custom-tailored and re-optimized for each individual primary antibody target. This lack of platform modularity hinders the rapid, comparative testing of diverse targeting candidates and severely limits the clinical translation of antibody-directed nanocarriers [24].

While several ‘indirect’ targeting strategies have been explored to bypass these issues – most notably the avidin-biotin system - they present fundamental biological and technical hurdles. Avidin, a highly stable tetrameric glycoprotein, often exhibits a high degree of non-specific binding in vivo due to its basic isoelectric point and glycosylation. Furthermore, the extreme affinity of the avidin-biotin interaction can hinder the controlled release of therapeutic payloads. Most critically, avidin-based methods still require the chemical biotinylation of the primary antibody, a step that adds technical complexity and can impair the antibody’s binding affinity [25]. Other indirect linkers, such as bacterial Protein A or G, raise significant concerns regarding potential immunogenicity and immunotoxicity, necessitating complex protein engineering to ensure safety for clinical applications [26,27].

To overcome these limitations, we leveraged the fundamental immunologic interaction between primary and secondary antibodies. Primary antibodies, such as immunoglobulin G (IgG), are generated to specifically recognize and bind to target antigens via their variable (Fab) regions. In contrast, secondary antibodies are designed to recognize conserved regions of primary antibodies, specifically the constant (Fc) domain, allowing them to bind universally across antibodies of the same species and isotype. For example, an anti-mouse IgG secondary antibody can selectively bind to the Fc domain of mouse-derived primary IgG in a rapid and non-covalent manner [28]. By utilizing secondary antibodies as highly stable, pre-oriented linkers on the liposome surface, we developed Universal Particles (UPs). Unlike traditional covalent immunoliposomes, this architectural arrangement supports an Fc-mediated, “tail-on” capture mechanism. This site-specific orientation projects the primary antibody’s active Fab domains directly outward into the aqueous phase, maximizing steric accessibility, facilitating multivalent target engagement, and completely bypassing the need to chemically modify the primary targeting antibody. In this study, we demonstrate that these UPs, formulated as liposomes displaying a secondary antibody via conjugation of PEGylated-phospholipid linkers, enable simple antibody modification of the liposomal surface. The targeting capability of UPs was first assessed by fluorescence-based quantification of liposome-protein interaction between anti-GFP liposomes and free GFP. To evaluate the potential for antibody-directed cell-specific delivery using UPs, we utilized a rat αCD31 antibody. In vivo experiments demonstrated that UPs modified via anti-rat αCD31 via antirat secondary antibody are specifically accumulated in the lungs, consistent with targeting of CD31-expressing endothelial cells in the pulmonary vasculature [29–31]. Moreover, we targeted the Panc02 mouse pancreatic tumors by modifying UPs to interact with their cell-specific markers Muc1 and Muc4 [32–34].

## 2 Materials and methods

### 2.1 Liposome preparation

Liposomes were prepared using the ethanol injection method. First, a 50 mM total lipid mixture of hydrogenated soybean phosphatidylcholine HSPC (Lipoid, Germany, Cat# 525600), cholesterol (Sigma-Aldrich, Israel, Cat# C8667), 1,2-distearoyl-sn-glycero-3-phosphoethanolamine-N-[methoxy(polyethylene glycol) − 1000] (ammonium salt) (DSPE-PEG1000) (Biopharma PEG, USA, Cat# MF001096), and 1,2-distearoyl-sn-glycero-3-phosphoethanolamine-N-[acetyl-(polyethylene glycol)-2000-O-acetic acid] (DSPE-PEG2000-COOH) (sodium salt) (Biopharma PEG, USA, Cat# LP096017), in molar percentages of 55:40:2.5:2.5, was dissolved in absolute ethanol and heated to 65 °C. Then, the lipid suspension was injected into an aqueous solution of Phosphate Buffered Saline (PBS) in a 1:9 volume ratio (ethanol: PBS) to form multilamellar liposomes. The liposome mixture was then down-sized by stepwise extrusion through 200, 100, and 100 nm pore size polycarbonate membranes (LIFEGENE, Israel, Cat# 110606 and 110,605, respectively) using a LIPEX extruder (Northern Lipids, Canada) at 65 °C, with a maximum working pressure of 15 bar to obtain homogeneous 100 nm liposomes. For fluorescently labeled liposomes preparation 1,2-distearoyl-sn-glycero-3-phosphoethanolamine (DSPE) (Lipoid, Germany, 565400) was mixed with Cyanine5 NHS ester (Cy5) (Lumiprobe, USA, Cat# 43020) in a 1:1 M ratio (DSPE: dye) to yield Cy5-DSPE-synthesized lipids. Then, the labeled lipid was added to the lipid mixture at a 0.2% molar ratio, achieving labeled liposome compositions of HSPC:Cholesterol:DSPE-PEG2000-COOH:DSPE-PEG1000:DSPE-Dye in molar percentages of 55:40:2.3:2.5:0.2, respectively.

### 2.2 Liposome characterization

Liposomal hydrodynamic diameter (nm), particle size distribution, polydispersity index (PDI), *Z*-average size (Fig. S1A), and particle concentration (particles/ml) were measured in PBS using a Zetasizer Ultra (Malvern, United Kingdom).

### 2.3 Universal particles (UPs) preparation

Anti-rat, anti-mouse antibodies (Jackson ImmunoResearch Laboratories Inc., USA, Cat# 115–005-008, 112–005-008) were cross-linked to the surface of liposomes by a two-step procedure. First, the free carboxyl group of the linker lipid incorporated in the liposomes was activated with N-(3-dimethylaminopropyl)-N′-ethyl carbodiimide hydrochloride (EDC) (Sigma-Aldrich, Cat# 03450) and N-hydroxysulfosuccinimide sodium salt (Sulfo-NHS) (Tzamal D-Chem Laboratories Ltd., Israel, Cat# FH24507), a well-known carbodiimide conjugation reaction described previously [35]. 1:25 ratio of DSPE-PEG2000-COOH to Sulfo-NHS and 1:10 M ratio of DSPE-PEG2000-COOH to EDC were used. Optimal activation with EDC and Sulfo-NHS occurs at pH 4.5–7.2 (EDC reactions often at pH 4.7–6.0). Therefore, we formulated the liposomes in PBS buffered at pH 6 using 100 mM HCl. The reaction occurred under mixing (450 rpm) at 4 °C for one hour. The liposome solution was dialyzed against PBS (pH 6; 1:1000 volume ratio) using a 12–14 kDa dialysis membrane (Repligen, USA, Cat# 132700) at 4 °C overnight to remove excess reagents.

Next, the antibodies were covalently conjugated to the lipids through displacement of sulfo-NHS groups by antibody amines. By mixing 100 nm empty NHS-liposomes with the antibody solution, the pH of the liposome reaction was adjusted to 8.4 with 1 M sodium bicarbonate, and the reaction was incubated for two hours at 450 rpm and maintained at 4 °C at a 1:30 (liposome particle: antibody molecule) ratio to synthesize immunoliposome / universal particle (UPs). The non-conjugated antibody was removed by Tangential Flow Filtration (TFF). To functionalize the surface of UPs with primary targeting antibodies, the selected antibodies were incubated with the UPs at 4 °C for 1 h, allowing non-covalent Fc-mediated binding to the surface-conjugated secondary antibodies.

### 2.4 Tangential flow filtration (TFF)

Following the conjugation of antibodies to liposomes, Tangential Flow Filtration (TFF) was employed to effectively remove unbound antibodies from the liposome suspension. A Spectrum® MidiKros Hollow Fiber Filter mPES / 0.05 μm pore size (Repligen, USA, Cat# C02-P50U-10-S) was used for this purpose. The liposome-antibody mixture was circulated tangentially across the membrane surface, creating a pressure gradient that allowed molecules smaller than the membrane’s molecular weight cut-off (MWCO) to pass through the membrane as permeate (free antibodies). At the same time, the larger liposomes (with conjugated antibodies) were retained in the retentate. The filtration process was performed at a controlled flow rate and transmembrane pressure (TMP), with a flow rate of 70 mL/min and a TMP maintained between 1 and 2 bars. To ensure thorough removal of unbound antibody, a diafiltration step was performed by adding Phosphate-Buffered Saline (PBS), pH 7.4, equivalent to 10 diafiltration volumes to the retentate at a controlled rate, while maintaining a constant retentate volume. This process effectively washed away the unbound antibody and other small molecules. The retentate volume was maintained at approximately the initial volume before proceeding with the diafiltration step to remove unbound antibody. The final retentate, containing the purified antibody-conjugated liposomes, was collected, and its concentration (particles/ml) and size diameter (nm) were determined by Zetasizer Ultra (Malvern, United Kingdom) as described above. The purified immunoliposomes were then characterized further to determine the antibody conjugation efficiency by using the tryptophan quantification assay (TQA).

### 2.5 Tryptophan quantification assay (TQA)

The efficiency of antibody conjugation to liposomes was determined using a Tryptophan Quantification Assay (TQA) based on the intrinsic fluorescence of tryptophan residues in the antibody structure. This method leverages that tryptophan is a primary contributor to protein fluorescence when excited in the UV range (~280 nm) and emits at ~350 nm. The Tryptophan Emission Spectrum (Fig. S1B) shows the range of light emitted when tryptophan is excited, which is typically between 300 and 500 nm, with a peak at around 350 nm in aqueous solution. Following the purification of antibody-conjugated liposomes via TFF, a 0.1 mL aliquot of the purified sample was directly added to a UV Microplate 96-well (Greiner Bio-One GmbH, Austria, Cat#655801). Fluorescence measurements were performed using a spectrofluorometer (Tecan i-control, 2.0.10.0, Infinite 200Pro, S# 2010011151). Samples were excited at a wavelength of 280 nm, and the emission spectrum was recorded at 350 nm. To generate a standard curve for antibody quantification, the same initial amount of a sample of the unconjugated antibody that was used in the conjugation reaction was serially diluted in a naked liposome solution to create dilutions of 100%, 10%, 1%, and 0.1%, each with a final volume of 0.1 mL, which were then measured under the same fluorescence settings. The antibody concentration in the antibody-conjugated liposome was determined by interpolating its fluorescence values against this standard curve. A linear regression calibration curve was generated by plotting the background-subtracted tryptophan fluorescence intensity (y) against known relative antibody input percentage, with the starting antibody input defined as the 100% reference (x) (Fig. S1C). The resulting equation was:

$$y=4588.2x+908.44\;(R^{2}=0.9992)$$

For each experimental sample, the background-subtracted fluorescence intensity (I_sample_) was converted to the corresponding antibody-input-equivalent (x_sample_) by rearranging this linear regression equation:

$$x_{\text{sample}}=\frac{I_{\text{sample}-908.44}}{4588.2}$$

Net primary antibody recruitment (Δx) estimated as the difference between the antibody-input-equivalent values obtained for αGFP-mAb-Lipo and mAb-Lipo:

$$\Delta x=x_{\alpha \text{GFP}–\text{mAb}–\text{Lipo}}-x_{\text{mAb}–\text{Lipo}}=150.82\%-86.61\%=64.21\%$$

The average antibody molecule number per liposome was then calculated by converting the antibody concentration to the number of antibody molecules using Avogadro’s number (6.022 × 1023 molecules/mol) and dividing the number of antibody molecules by the number of liposomes.

### 2.6 Cell culture

Panc02 cell line, a murine model for pancreatic ductal adenocarcinoma (PDAC), was kindly provided by Prof. Israel Vlodavsky (Technion, Haifa, Israel). Cells were grown in Dulbecco’s Modified Eagle’s Medium (DMEM) (D5796; Sigma-Aldrich, Cat# 30–2002) supplemented with 10% (*v*/v) fetal bovine serum (FBS) (Gibco, Cat# 26140079) and 1% (*v*/v) penicillin (10,000 units / mL), streptomycin (10 mg / mL; Pen-Strep) (Biological Industries, Israel, Cat# 030311B). Cells were cultured at 37 °C in a humidified atmosphere incubator containing 5% CO2, a fresh medium was added every 2–3 days. Dulbecco’s Phosphate Buffered Sa-line (PBS) (Sigma-Aldrich, Cat# BSS-1005-B) was used for cell wash. Trypsin-EDTA (SM2003C; Merck Millipore) was used for cell dissociation. Cells were passaged in culture for no more than 2 months after being thawed from authentic stocks.

### 2.7 Indirect flow cytometry analysis

Flow cytometry was used to determine the expression of Muc1 and Muc4 on Panc02 cells. Briefly, Panc02 cells were harvested, washed with PBS containing 2% FBS (FACS buffer), and resuspended at a concentration of 0.5 × 10^6^ cells/mL in FACS buffer. For indirect staining, cells were incubated with the primary antibodies: anti-Muc1 mouse monoclonal antibody (Santa Cruz, Cat# sc-53,381) at a dilution of 1:200 and anti-Muc4 mouse monoclonal antibody (Santa Cruz, Cat# sc-33654) at a dilution of 1:200. Cells were incubated with the primary antibodies for 30 min at 25 °C. Following incubation with the primary antibodies, cells were washed twice with FACS buffer to remove unbound antibody. Subsequently, cells were incubated with the secondary antibody: Alexa Fluor® 647-conjugated Anti-Mouse IgG (Jackson ImmunoResearch Laboratories, Inc., Cat# 715–605-150) at a dilution of 1:500 in FACS buffer for 30 min at 25 °C in the dark. After incubation with the secondary antibody, cells were washed twice with FACS buffer to remove unbound secondary antibody and then resuspended in FACS buffer for flow cytometric analysis. Flow cytometry was performed using a BD® LSR II Flow Cytometer and FACSDiva software (BD Biosciences). Data were collected for 10,000 events per sample. Analysis was performed using FlowJo software (BD Biosciences) to determine the percentage of positive cells and the mean fluorescence intensity (MFI) for Alexa Fluor® 647, which correlates with the expression levels of Muc1 and Muc4.

### 2.8 Confocal microscopy

For uptake experiments, Panc02 cells were seeded onto a 24-well glass-bottom plate with high-performance #1.5 cover glass (0.170 ± 0.005 mm) with a Black frame (Cellvis, USA, Cat# P24-1.5H-N) at a density of 3 × 10^5^ cells per well and allowed to adhere overnight. Cells were then incubated with the fluorescently labeled-modified UPs (targeting Muc1 and Muc4). Following incubation, the cells were washed three times with PBS to remove any unbound UPs. To visualize the cells and potentially specific cellular compartments, cells were optionally stained with Hoechst 34580 (Sigma-Aldrich, Cat# 63493) for nucleus staining according to the manufacturer’s instructions. Confocal microscopy was performed using a Zeiss LSM 710 confocal microscope system equipped with a 63× oil immersion objective. Cy5 fluorescence associated with UPs was excited using a 640 nm laser source, and emission was collected at 680 nm. Image analysis was performed using ZEN Microscopy Software (ZEISS, Blue Edition). The uptake of UPs was assessed by visually inspecting the presence of fluorescent UPs within the cellular boundaries.

### 2.9 Establishment of the Panc02 tumor model

Male C57BL/6 mice, aged 8 weeks, were obtained from Envigo. Mice were housed under specific pathogen-free (SPF) conditions with ad libitum access to food and water. All animal procedures were performed in accordance with institutional guidelines and approved by the Inspection Committee on the Constitution of the Animal Experimentation at the Technion (IL-038-03-2024). Panc02 cells were harvested, washed with sterile Phosphate-Buffered Saline (PBS), and resuspended at a concentration of 5 × 10^6^ cells/mL in PBS. 5 × 10^5^ cells were subcutaneously injected into the right flank of each mouse. Tumor growth was monitored twice weekly using calipers, and tumor volume was calculated using the formula $\frac{\text{Length}\;\times {\;\text{Width}}^{2}}{2}$. Mice with tumors reaching a volume of approximately 300–400 mm^3^ were selected for biodistribution studies.

### 2.10 Preparation and administration of targeted UPs

UPs were prepared as described above. These UPs were modified by secondary-primary antibody affinity reaction with antibodies against CD31 (InVivoMAb anti-mouse CD31, BIOXcell, USA, Cat# BE0377) or antibodies against Muc1 and Muc4, as described above, to target lungs or tumors, respectively. The UPs were fluorescently labeled with Cy5 conjugated to the liposome membrane for in vivo imaging as described above. Mice bearing established Panc02 tumors were randomly divided into experimental groups. The fluorescently labeled UPs were administered intravenously via the tail vein at a dose of 10^9^ to 10^12^ particles per 25 g mouse weight in a volume of 200 μL of sterile PBS.

### 2.11 In vivo imaging

At predetermined time points post-injection (4 h for CD31 experiment and 48 h for Panc02 tumor experiment), mice were anesthetized with 100 mg/kg ketamine and 10 mg/kg xylazine and then perfused by cutting the aorta and infusing PBS through the left ventricle of the heart. The tumor and major organs (liver, spleen, kidneys, lungs, heart) were then excised. Fluorescence imaging was performed using an IVIS Spectrum CT imaging system (PerkinElmer) and a VECTor7 preclinical imaging system (MILabs), with excitation/emission wavelengths optimized for Cy5. Images were acquired and specific regions of interest (ROIs) were drawn around the tumor and major organs to quantify fluorescence signal intensity using the system’s Living Image® software (PerkinElmer).

### 2.12 Data analysis

Quantitative fluorescence data from in vivo imaging were analyzed using GraphPad Prism 9. Values are expressed as mean ± SEM. Statistical differences between two groups were determined using an independent samples *t*-test. A *p*-value of less than 0.05 was considered statistically significant.

## 3 Results

### 3.1 Synthesis and physicochemical characterization of UPs

**Fig. 1:**
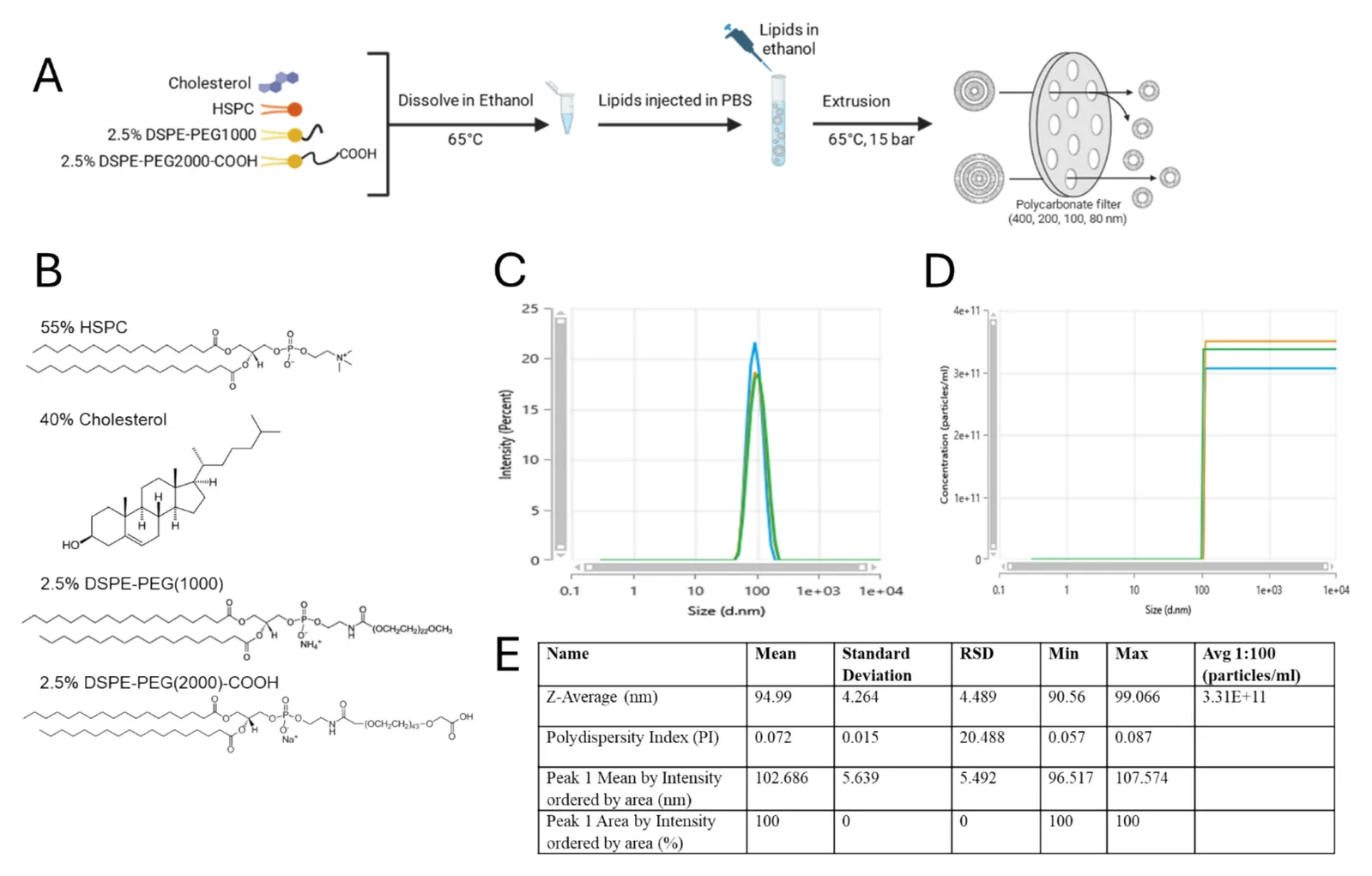
Liposome preparation and characterization A, Illustration of ethanol injection and extrusion method. B, Lipid composition of the liposomes. C, The diameter measurement of liposomes. D, Concentration measurement of liposomes. E, Physicochemical characterization of liposomes.

For the UPs synthesis, liposomes were prepared using an ethanol injection and extrusion method, as illustrated in Fig. 1A. The core lipid composition of the liposomes, includes 55% HSPC, 40% cholesterol, 2.5% DSPE-PEG1000, and 2.5% DSPE-PEG2000-COOH (Fig. 1B). Briefly, lipids were dissolved in ethanol at 65 °C, injected into PBS at 65 °C, and subsequently extruded through polycarbonate filters (400 nm, 200 nm, 100 nm, and 80 nm pore sizes) at 65 °C and 15 bar to achieve a narrow size distribution. The physicochemical properties of the synthesized liposomes were then characterized. The liposomes exhibited a Z-Average hydrodynamic diameter of 94.99 ± 4.264 nm and a low polydispersity index (PDI) of 0.072 ± 0.015 (Fig. 1C and E). Moreover, the rapid decay of the correlation coefficient, reaching baseline in approximately 10^3^ μs, is characteristic of small particles undergoing fast Brownian motion, validating the low Z-average size and demonstrating successful fabrication and stability (Fig. S1A). A total lipid concentration of 50 mM yielded a particle concentration of 3.31 × 10^13^ particles/mL, as measured using Zetasizer Ultra (Fig. 1D and E). These results confirm the successful formation of a high concentration of uniform liposomes, with optimal size and homogeneity for in vivo applications.

**Fig. 2:**
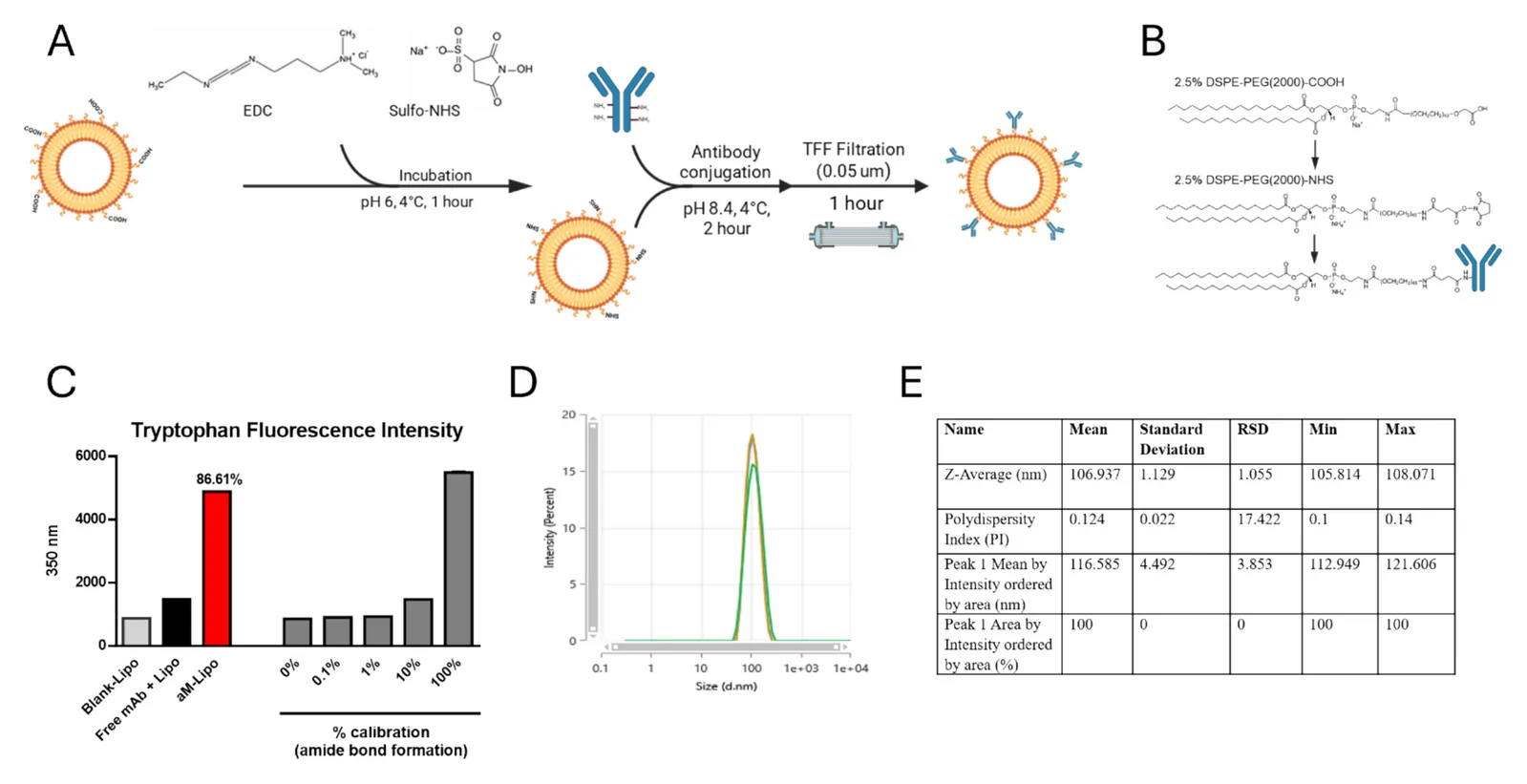
UPs preparation and characterization A, illustration of conjugation reaction. B, covalent conjugation of the antibodies to the activated NHS-lipid. C, quantified antibody using a TQA. D, The diameter measurement of UPs. E, Physicochemical characterization of UPs.

The conjugation of antibodies to the synthesized UPs was achieved via a two-step carbodiimide chemistry, as schematically depicted (Fig. 2A). This process involved the activation of the carboxyl groups of DSPE-PEG2000-COOH linker lipids within the liposome membrane using EDC and Sulfo-NHS. The activation reaction was performed at pH 6. Following activation, the antibodies were covalently conjugated to the activated NHS-liposomes through the displacement of sulfo-NHS groups by antibody amines (Fig. 2B). The non-conjugated antibody was subsequently removed by TFF using a 0.05 μm membrane. The efficiency of antibody conjugation was quantified using a TQA. This assay utilizes the intrinsic fluorescence of tryptophan residues in the antibody structure, which emits light at approximately 350 nm upon excitation at 280 nm. The Tryptophan Emission Spectrum (Fig. S1B) shows the range of light emitted (300 to 500 nm) when tryptophan is excited, with a peak around 350 nm, making it an effective tool for detecting the antibody bound to the liposome. The antibody concentration in each sample was subsequently calculated from a linear regression calibration curve generated using known antibody inputs, as described in the Methods section.

The purification efficacy of TFF in removing unbound antibody was confirmed by measuring the tryptophan emission peak at 350 nm in the permeate fraction (Fig. S1D), with the stably bound antibody remaining efficiently retained after the wash process (Fig. S1E). As shown in Fig. 2C, the fluorescence intensity of the mouse antibody-conjugated UPs (mAb-Lipo) was significantly higher than that of blank liposomes (Blank-Lipo) and free antibody (Free mAb), indicating successful antibody attachment. A tryptophan fluorescence calibration curve generated from serially diluted unconjugated antibody in a blank liposome suspension (Fig. S1C) was used to determine the antibody-input-equivalent value of the UPs. The conjugation efficiency was calculated to be 86.61% (Fig. 2C), indicating a high yield of secondary-antibody attachment to the UPs. This conjugation reaction was carried out by mixing the activated liposomes with the antibody solution at a 1:30 (liposome particle: antibody molecule) ratio. 86.61% indicates that ~26 antibodies are conjugated per liposome. The particle size increased from 94.99 ± 4.264 for naked liposomes (Fig. 1C and E) to 106 ± 1.129 nm after mAb conjugation (Fig. 2D and E). This was accompanied by an increase in the polydispersity index (PDI) from 0.072 ± 0.015 to 0.124 ± 0.022, indicating increased heterogeneity following surface modification, while remaining within an acceptable range for nanoparticle formulations.

### 3.2 UPs engineered for specific binding to soluble GFP demonstrate molecular recognition

**Fig. 3:**
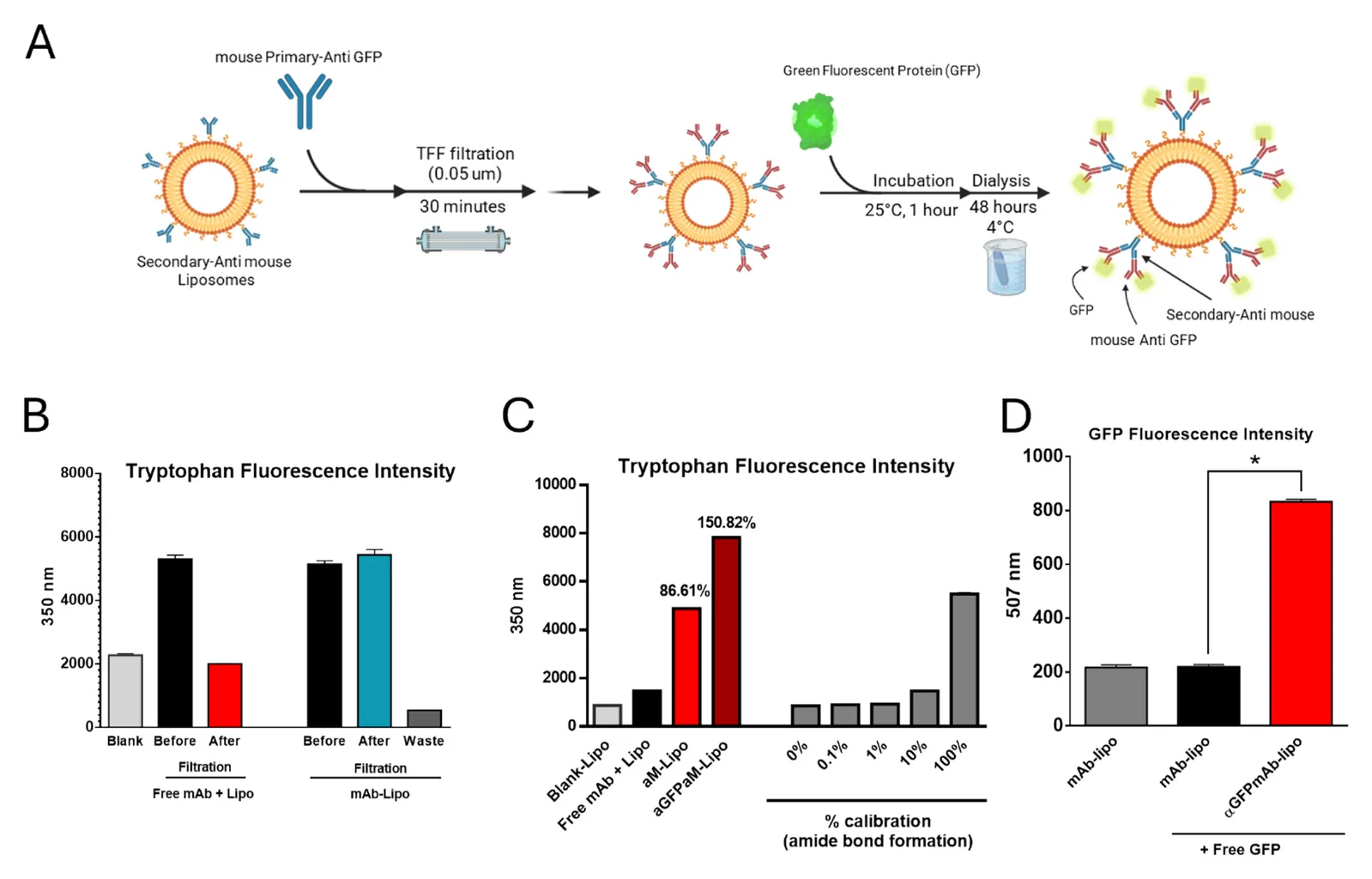
Molecular recognition of GFP by the engineered UPs A, Illustration of engineered UPs and their interaction with free GFP. B, Antibody conjugation stability after the TFF purification step. C, TQA of antibody binding to UPs. Bars represent blank liposomes, liposomes with free antibody (free mAb + lipo), liposomes functionalized with secondary antibody (mAb-lipo), and liposomes functionalized with secondary antibody and bound primary anti-GFP antibody (aGFP–mAb-lipo). D, detection of the GFP interacting with engineered UPs. * *p* < 0.0001, relative to mAb-lipo.

To assess the functional ability of the antibody-conjugated UPs to specifically recognize and bind their target antigen, a molecular assay was performed using UPs modified with anti-GFP antibodies. The experimental scheme for this functionality assessment, involving the sequential binding of secondary anti-mouse antibodies to the liposomes, followed by the primary mouse anti-GFP antibody (αGFP), and subsequent incubation with purified free GFP, is outlined in Fig. 3A. The successful conjugation of the secondary anti-mouse antibodies to the liposomes was confirmed by TQA. Fig. 3B displays the Tryptophan fluorescence intensity (emission at 350 nm, indicative of antibody presence) at various stages of the antibody conjugation process. The blank liposomes exhibited a baseline fluorescence signal. Unbound antibody (Free mAb + Lipo) and antibody-conjugated liposomes (mAb-Lipo) demonstarted a higher fluorescence intensity compared to the blank, confirming the presence of free or conjugated antibody in the solution before the TFF washing step. After the TFF purification step, a reduction in fluorescence intensity of Free Ab + Lipo was observed, indicating that the unbound antibody was effectively washed away. Conversely, tryptophan fluorescence intensity remained high for the mAb-Lipo after TFF purification, indicating successful antibody attachment. Moreover, the weak signal in the waste fraction indicates the low percentage of the unbound antibody in the conjugation reaction.

To evaluate the non-covalent recruitment of the primary targeting antibody, the antibody-input-equivalent values of mAb-Lipo and αGFP-mAb-Lipo were estimated using the linear calibration equation described in the Methods, yielding 86.61% and 150.82%, respectively (Fig. 3C). Net primary antibody recruitment was estimated as the difference between these values (150.82% − 86.61%), corresponding to 64.21 antibody-input-equivalent percentage points relative to the starting antibody input reference. This confirmed the successful functionalization of the UPs with the specific anti-GFP antibody for subsequent antigen-binding assays.

The GFP Emission Spectrum (Fig. S1F) peaks at around 507 nm, and unbound GFP was efficiently removed via TFF (Fig. S1G), confirming the successful removal of free GFP. The specific binding of GFP to the anti-GFP-functionalized UPs was then directly evaluated by monitoring GFP’s characteristic fluorescence emission at 507 nm. As presented in Fig. 3D, the GFP fluorescence intensity was significantly higher (*p* < 0.0001) for UPs conjugated with both secondary and primary anti-GFP antibodies (αGFP-mAb-Lipo) compared to blank liposomes (Blank-Lipo) or liposomes conjugated only with secondary antibodies (mAb-Lipo). This marked increase in the GFP-specific signal confirms the successful and specific molecular recognition and binding of soluble GFP by the engineered UPs. To evaluate the stability of this non-covalent, Fc-mediated linkage under physiological conditions, UPs incubated in 10% FBS for 48 h maintained their targeting capability, with the resulting GFP fluorescence intensity being significantly higher than that of naked liposomes under the same conditions. This robust resistance to competitive displacement by endogenous serum proteins was evident, as serum-exposed particles (UPs-FBS) showed no significant loss of targeting functionality compared to serum-free controls (UPs-PBS), as illustrated in Fig. S1H.

### 3.3 Enhanced in vitro cellular uptake of anti-Muc1+4-functionalized UPs in Panc02 cells

**Fig. 4:**
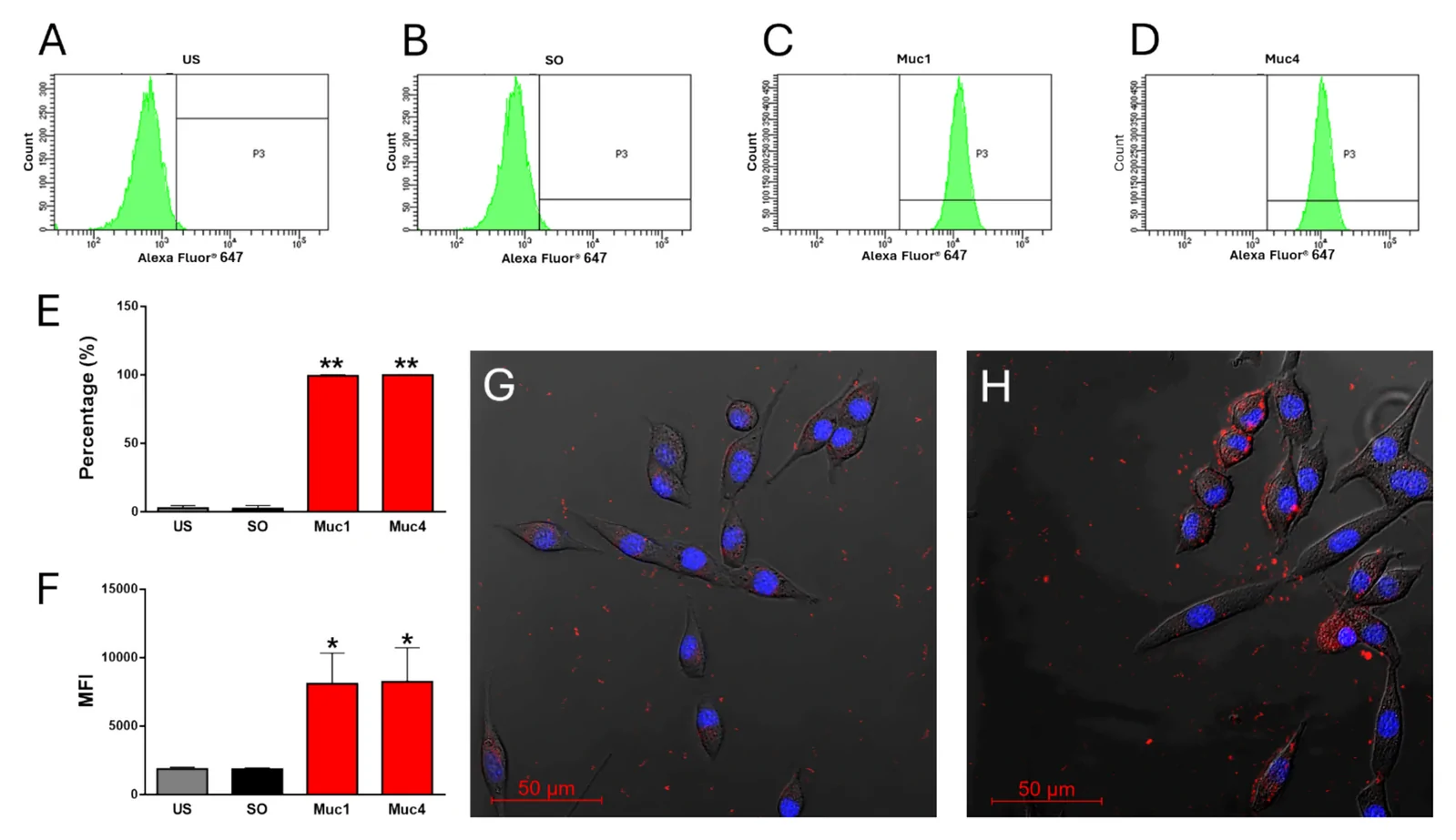
Evaluation of Muc1 and Muc4 antigen expression on Panc02 cells and in vitro cellular uptake of targeted UPs. A-D, representative analytical flow-cytometry histograms of Panc02 cells; unstained (US), secondary-only (SO), Muc1, Muc4 expression profiles, respectively. E-F, percentage of positive cells and Mean Fluorescence Intensity (MFI), respectively, derived from the flow cytometry data (Mean ± SEM). G-H, representative confocal images of Panc02 cells incubated with Cy5 - labeled UPs without (G) or with (H) Muc1 + Muc4 functionalization. Composite images show nuclei stained with Hoechst (blue) and UPs detected via Cy5 fluorescence (red). *p < 0.05, **p < 0.001, relative to SO. (For interpretation of the references to colour in this figure legend, the reader is referred to the web version of this article.)

We base our targeting on molecular analysis. We selected primary antibodies based on cell surface profile markers that are specifically expressed on PDAC cells, as these markers represent promising candidates for targeted delivery evaluation. Following a screen of commercial antibodies, we have identified cell-surface markers for targeting/separating pancreatic adenocarcinoma cells from healthy cells. Two putative cell-surface markers (Muc-1, Muc-4) were confirmed by analytical flow cytometry, with representative histograms showing distinct population shifts compared to unstained and secondary-only controls (Fig. 4A–D). Quantitative analysis confirmed that these target antigens exhibit exceptionally high expression levels, with 99.5% of cells positive for Muc1 and 99.9% positive for Muc4 (Fig. 4E), accompanied by a corresponding robust increase in mean fluorescence intensity (MFI) (Fig. 4F).

To validate our choice of Muc1 and Muc4 as therapeutic targets, we analyzed their expression and prognostic relevance in pancreatic lesions and various tumors. A Kaplan-Meier survival analysis (Fig. S2A and B) revealed that high expression of Muc1 and Muc4 is associated with a significantly poorer prognosis, suggesting their importance in disease progression. Furthermore, semi-quantitative analysis across different stages of pancreatic neoplasia confirms the strong expression of Muc1 and Muc4 in PanIN III and Pancreatic Cancer [34]. Supporting this, box plots of Muc1 and Muc4 gene expression across tissue/tumor types show high expression in Panc02 pancreatic tumor model (Fig. S2C and D). To evaluate the targeting capability of antibody-functionalized and Cy5-labeled UPs in vitro, confocal microscopy was performed on Panc02 cells. As depicted in Fig. 4G, control UPs (lacking anti-Muc1+4 antibodies) exhibited limited association or uptake by the Panc02 cells (red signal), with minimal intracellular localization observed. In contrast, Fig. 4H clearly shows a significant enhancement in cellular uptake of the UPs functionalized with antibodies targeting Muc1 and Muc4 (red signal) by the Panc02 cells. A greater number of cells show association with the targeted UPs, and a substantial amount of the red fluorescent signal is visible within the cellular boundaries, indicating efficient internalization of the antibody-conjugated nanoparticles. These confocal microscopy findings strongly suggest that the anti-Muc1+4 antibodies displayed on the surface of the UPs effectively mediate increased cellular uptake by the target Panc02 cells in vitro.

### 3.4 Biodistribution of CD31-targeted UPs shows lung accumulation in viv

**Fig. 5:**
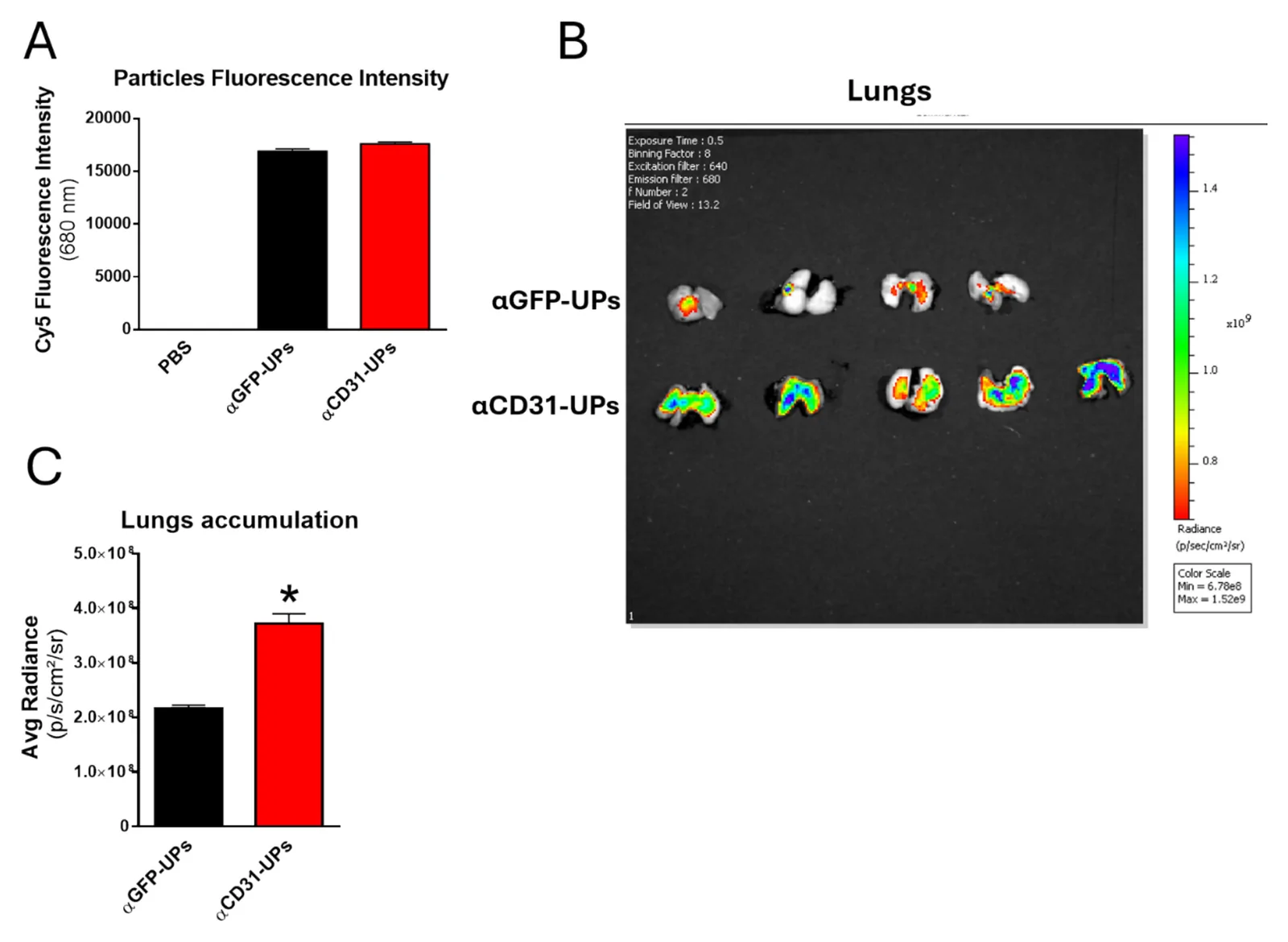
In vivo lung targeting by aCD31-UPs A, Cy5 fluorescence intensity of the particles. B, Fluorescence signal of the Cy5-labeled particles in the lungs by IVIS imaging system. C, The ROI quantitative fluorescence intensity analysis. *p < 0.0001, relative to αGFP-UPs.

To investigate the in vivo organ biodistribution and specific targeting of UPs to the lung vasculature, we prepared a CD31-UPs formulation (UPs functionalized with αCD31 antibodies) and a control formulation of anti-GFP UPs. Both formulations were labeled with Cy5, as described in the UPs preparation process in the Methods section, and a 200 μL volume of each formulation was administered intravenously via the tail vein into C57BL/6 mice. The major organs (lungs, liver, spleen, kidneys, heart) were harvested at the same 4-h time point, and fluorescence images were obtained immediately after tissue harvesting using an IVIS imaging system (Cy5: λex =640 nm; λem =680 nm). The quantitative fluorescence signal of the Cy5-labeled UPs allowed comparison of the overall signal intensity between the control αGFP-UPs and the αCD31-UPs. The similar intensity levels observed in this quantitative analysis for both αGFP-UPs and CD31-targeted UPs suggest a comparable initial labeling efficiency of the nanoparticles (Fig. 5A). The choice of CD31 as a targeting molecule is supported by the protein expression overview of PECAM1 (CD31) across a wide range of human organs (Fig. S2E). The bar chart confirms the dominant presence of this endothelial cell marker specifically in the lungs, providing the molecular rationale for the observed pulmonary tropism. However, a significantly increased fluorescence signal was observed specifically in the images of the lungs from the αCD31-UPs mice group compared to the lungs from αGFP-UPs mice group (Fig. 5B).

Moreover, quantitative analysis of the lung ROIs revealed a significantly higher fluorescence signal in the αCD31-UPs group compared to the αGFP-UPs group, *p* < 0.0001 (Fig. 5C), consistent with the enhanced fluorescence intensity visually observed in the corresponding organ image (Fig. 5B). These results indicate that the αCD31-UPs likely exhibit enhanced accumulation and targeting of the lung tissue compared to the non-targeted UPs (αGFP-UPs). The higher localized signal in the images suggests a more specific interaction and retention of the CD31-targeted UPs within the lungs.

These findings of enhanced pulmonary targeting were reproducible, as demonstrated by an independent biological replicate using a VECTor7 preclinical imaging system (MILabs). The replicate in vivo fluorescence images of excised lungs confirmed a significantly higher accumulation of fluorescence in the αCD31-UPs group compared to both the untreated (Cont) and non-targeted Liposome (Lipo) groups (Fig. S3A and B). Furthermore, minimal fluorescence was consistently observed in the kidneys across all groups in this replicate (Fig. S3C and D), reinforcing the limited renal clearance or accumulation of αCD31-UPs in the lungs and the consistency of the observed biodistribution pattern.

**Fig. 6:**
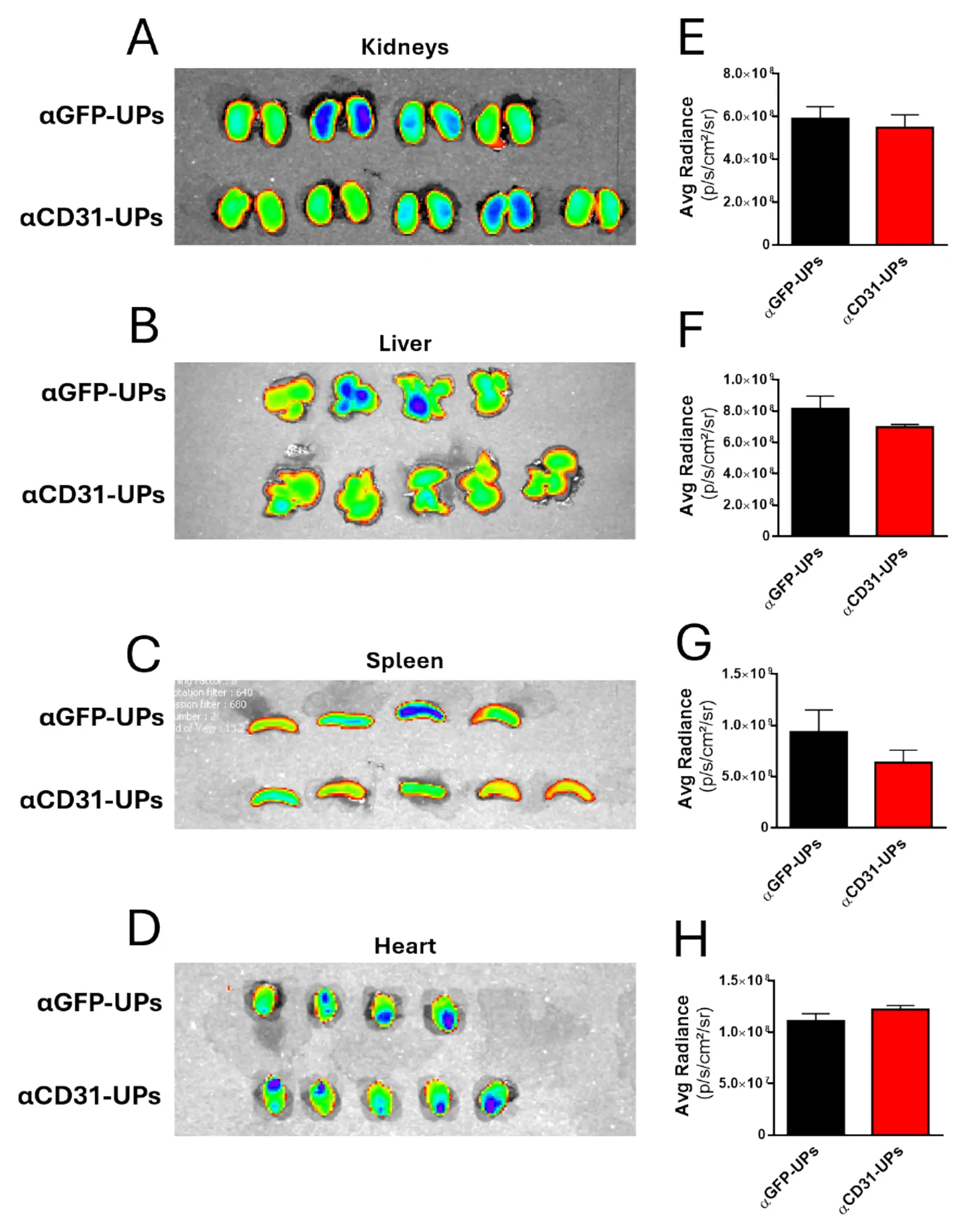
aCD31-UPs in vivo biodistribution in major organs A-D, Fluorescence signal of the Cy5-labeled particles in major organs by IVIS imaging system. E-H, The ROI quantitative fluorescence intensity analysis.

Beyond the specific accumulation observed in the lungs, the bio-distribution of αCD31-UPs in other major organs was also assessed and compared to αGFP-UPs. Fig. 6A-D displays the in vivo fluorescence images of excised kidneys, liver, spleen, and heart, respectively, from mice administered with either αGFP-UPs or αCD31-UPs. Both the αGFP-UPs and the αCD31-UPs showed prominent accumulation in organs typically involved in nanoparticle clearance, such as the liver and spleen.

Quantitative analysis of the fluorescence intensity within defined regions of interest (ROIs) for these organs (Fig. 6E-H) revealed no significant differences in the accumulation profiles between the αCD31-UPs and the αGFP-UPs. These comparable quantitative values across the major organs indicate that while CD31 targeting effectively enhanced accumulation in the lungs, it did not significantly alter the general biodistribution pattern or accumulation levels in these other highly perfused organs, suggesting that the CD31-mediated targeting effect is specific to the pulmonary vasculature and does not broadly redirect UPs away from or toward other major organs.

### 3.5 Dual Muc1+4-targeted UPs demonstrate biodistribution and tumor accumulation in a murine Panc02 pancreatic tumor model in vivo

**Fig. 7:**
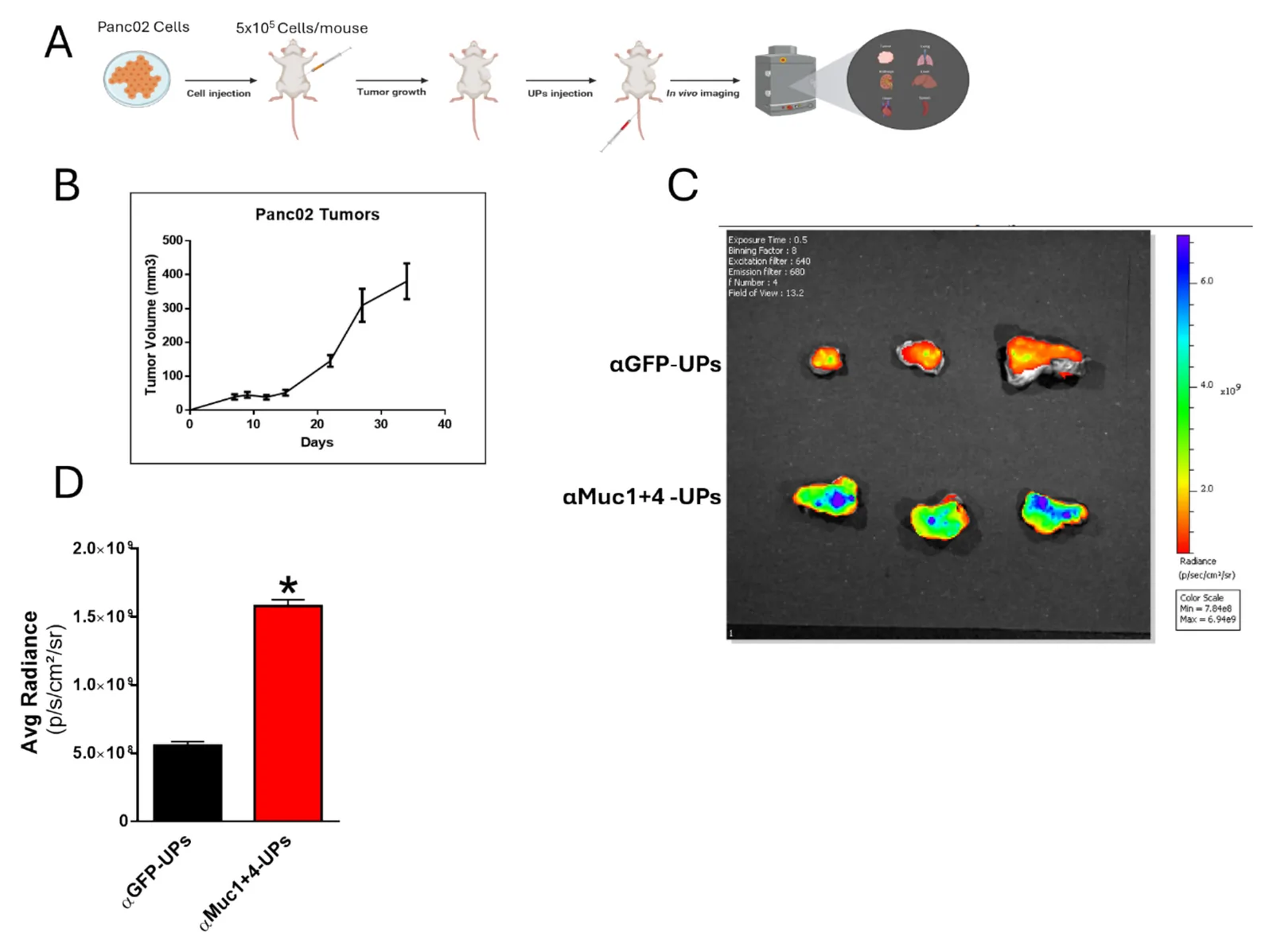
In vivo Pancreatic tumor targeting by αMuc1+4-UPs A, Subcutaneous tumor model timeline. B, Tumor volume measurements. C, Fluorescence signal of the Cy5-labeled particles in Panc02 tumors by IVIS imaging system. D, The ROI quantitative fluorescence intensity analysis. *p < 0.0001, relative to αGFP-UPs.

To evaluate the in vivo targeting and biodistribution of UPs toward pancreatic tumors, a murine Panc02 tumor model was established by subcutaneous injection of 5 × 10^5^ cells/mouse (Fig. 7A), and the tumor growth was monitored using a caliper. Tumors were successfully established, as demonstrated by a progressive increase in tumor volume over the course of the experiment (Fig. 7B). When tumors reached ~400 mm3, intravenous administration of the UPs was performed. 48 h post-injection, tumors and major organs were harvested for in vivo analysis.

The in vivo targeting efficacy of the Muc1+4-targeted UPs Muc1+4-UPs) was assessed using fluorescence imaging. As shown in Fig. 7C, the αMuc1+ 4-UPs group exhibited a strong fluorescence signal localized to the tumor site, indicating preferential accumulation of the targeted nanoparticles within the tumor. In contrast, the control group (αGFP-UPs) exhibited a significantly weaker signal, indicating less efficient tumor targeting.

Quantitative analysis of the fluorescence intensity within the tumor region of interest (ROI) (Fig. 7D) further confirmed the enhanced tumor accumulation of the αMuc1+4-UPs. The mean radiance (p/s/cm^2^/sr) in the tumors was 1.58 × 10^9^ ± 4.726 × 10^7^ for the αMuc1+4 UPs, which was significantly higher (*p* < 0.0001) than that of the αGFP-UPs, with a mean radiance of 5.567 × 10^8^ ± 2.90 × 10^7^. This statistically significant difference demonstrates the ability of the dual-targeted UPs to accumulate effectively within pancreatic tumors in vivo.

**Fig. 8:**
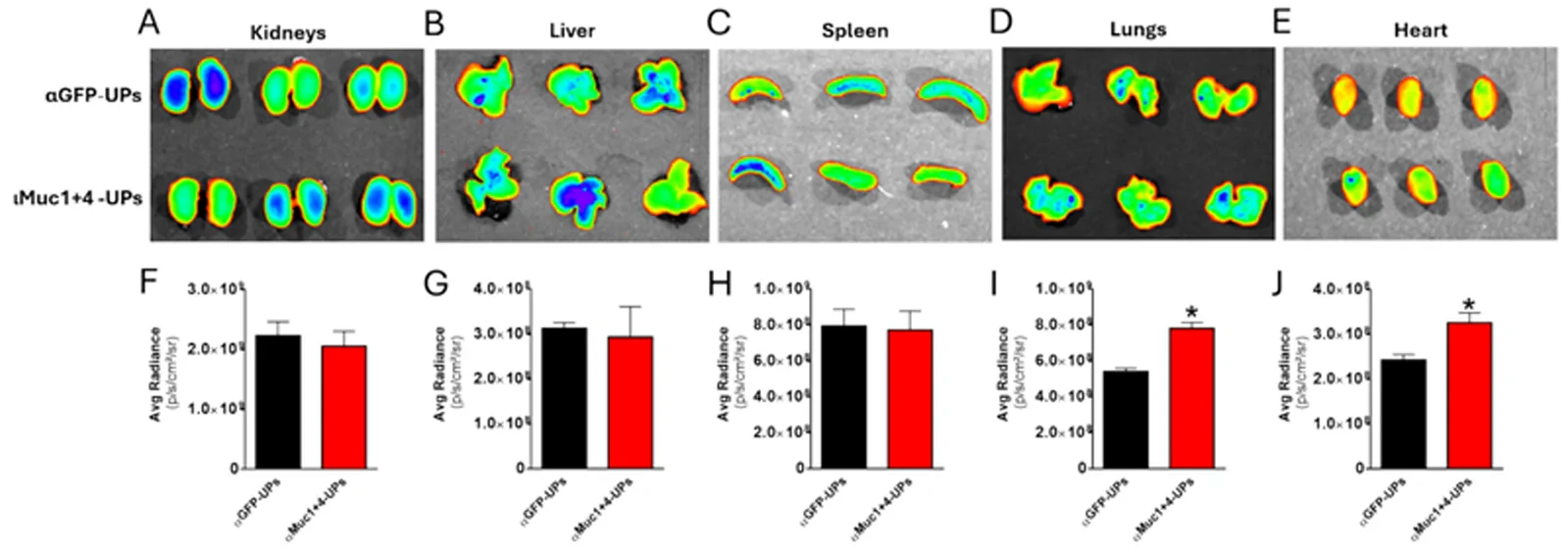
αMuc1+4-UPs in vivo biodistribution in major organs A-E, Fluorescence signal of the Cy5-labeled particles in major organs by IVIS imaging system. F-J, The ROI quantitative fluorescence intensity analysis. *p < 0.01, relative to αGFP-UPs.

To further understand the systemic distribution of the dual-targeted αMuc1+4-UPs beyond the tumor site, their accumulation in major organs was assessed and compared to αGFP-UPs in the murine Panc02 pancreatic tumor model. Visually, both αGFP-UPs and αMuc1+4-UPs exhibited significant accumulation in organs known for nanoparticle clearance, particularly the kidneys, liver, and spleen (Fig. 8A-C). However, a direct comparison of the fluorescence signal in these organs revealed no substantial differences between the targeted and untargeted UPs. In contrast, the biodistribution to lungs and heart (Fig. 8D and E) showed a different pattern. A visual comparison of the in vivo fluorescence images revealed an increased accumulation of the αMuc1+4-UPs compared to the αGFP-UPs control group. The signal appeared higher and more distributed throughout the lung and heart tissue in the targeted group. This suggests a potential secondary accumulation or retention of the dual-targeted UPs in the lungs, possibly due to interactions with lung tissue components.

This observation is quantitatively supported by the region of interest (ROI) analysis presented in Fig. 8F-J. Quantitative evaluation of the primary clearance organs (Fig. 8F–H) revealed comparable, statistically non-significant differences in nanoparticle accumulation in the kidneys, liver, and spleen between the αGFP-UPs and αMuc1+4-UPs groups, indicating that active tumor targeting did not broadly alter default metabolic clearance pathways. In contrast, statistically significant increases in accumulation were observed in both the lungs (Fig. 8I) and the heart (Fig. 8J) for the αMuc1+4-UPs group compared with untargeted controls, indicating secondary cardiopulmonary accumulation in addition to the enhanced tumor accumulation.

## 4 Discussion

The development of the UP platform offers a modular design of targeted liposomal nanocarriers. By attaching a secondary antibody to the liposomal surface through established carbodiimide chemistry, we overcome the complex process of chemical conjugation from the specific targeting ligand. This new architecture enables the rapid, robust, and stable attachment of different whole-molecule primary IgG antibodies via its conserved Fc region, effectively creating a “plug-and-play” system. This method expands the preclinical utility and modular design of immunoliposomes by enabling researchers and clinicians to rapidly exchange targeting ligands without requiring the re-optimization of complex surface chemical reactions for each new antibody. Beyond the simplicity of preparation, the UP platform provides a critical mechanistic advantage regarding antibody orientation. In conventional covalent immunoliposomes, chemical conjugation typically occurs randomly, leading to a random orientation in which many Fab regions may be ‘buried’ or face the liposome bilayer [21,36]. Such random packing introduces significant steric hindrance and reduces the number of functional binding sites. In contrast, the UP platform utilizes a secondary antibody to specifically ‘capture’ the primary antibody via its conserved Fc region. This site-specific, Fc-mediated binding promotes a ‘tail-on’ orientation, projecting the antigen-binding Fab domains outward and away from the liposome surface. This uniform display maximizes steric accessibility and enhances functional multivalency, ensuring that the primary antibodies attached to the UP, as estimated in our study, are optimally positioned for target engagement.

To appreciate the distinct advantages of the UP platform, it must be critically compared with other state-of-the-art indirect functionalization methods, such as the PAR28-PEG-lipo system reported by Hama et al. or the Annexin-A5-ZZ (Anx5ZZ) platform reported by Garnier et al. While Hama et al. rely on a low-temperature post-insertion method, the Anx5ZZ platform relies on a Ca^2+^-dependent Annexin-A5–phospholipid interaction that presents major bottlenecks. Specifically, because IgG antibodies are bivalent and bind multiple ZZ domains, Garnier et al. reported massive, uncontrolled vesicle aggregation (radii shifting from 54 nm to 1.5 μm) unless the adapter and targeting IgG are pre-complexed in solution prior to membrane adsorption. Furthermore, the binding of the Anx5 core depends entirely on millimolar concentrations of exogenous Ca2+, risking adapter desorption in physiological environments [37,38]. In contrast, our UP platform utilizes secondary antibodies covalently anchored to an integrated DSPE-PEG2000-COOH bilayer framework, providing a stable capture framework independent of calcium co-factors. This enables rapid, single-step “plug-and-play” functionalization without requiring the pre-complexation step used in the Anx5ZZ approach. To further contextualize the indirect targeting paradigm, the UP platform can be compared with the bispecific antibody (BsAb) multi-affinity strategy reported by Moles et al. Their elegant system recruits clinically approved PEGylated liposomal doxorubicin to leukemic cells using engineered BsAbs that simultaneously bind to PEG and to tumor-specific receptors (such as CD19, CD20, CD22, or CD38) [39]. While the bispecific antibody (BsAb) strategy by Moles et al. relies on engineered bispecific constructs tailored to the selected targeting receptor, the UP platform uses a universal secondary-to-primary antibody capture framework. This enables the incorporation of different off-the-shelf, commercially available IgGs without redesigning the nanoparticle platform for each target, thereby providing a complementary approach that may facilitate rapid adaptation and preclinical screening across different targeting antibodies.

Compared to established Fc-binding approaches utilizing bacterial proteins like Protein A or Protein G, the UP platform offers subclass specificity and potentially lower immunogenicity [26,27]. While Protein A and G provide broad binding across different species, they are bacterial derivatives that can be recognized as foreign by the immune system, potentially leading to accelerated clearance or inflammatory responses.

Despite these advantages, the use of whole IgG and exposed Fc domains presents challenges for clinical translation. Interaction with Fc receptors (FcγRs) on immune cells may trigger unintended uptake by the mononuclear phagocyte system, potentially accelerating clearance. Additionally, whole antibodies, especially those from different species, may be immunogenic upon repeated dosing [36]. While our short-term studies showed preferential target-site accumulation, future iterations could utilize Fc-engineered antibodies or recombinant mimics to minimize these risks and improve long-term safety.

A key translational challenge for the UP platform is the potential immunogenicity of animal-derived capture secondary antibodies. While the primary targeting antibody can be humanized, the capturing antibody must retain specificity for the human Fc region. If raised in non-human hosts (e.g., mouse or goat), these components can induce a human anti-species antibody response (e.g., Human Anti-Mouse Antibody and Human Anti-Goat Antibody) upon repeated clinical administration, leading to rapid therapeutic neutralization and clearance [40]. To address this anti-antibody response, future clinical iterations can replace animal-derived IgGs with humanized anti-human Fc monoclonal antibodies, low-immunogenic recombinant fragments (scFvs or humanized nanobodies), or synthetic Fc-binding peptides such as cyclic FcIII [41,42]. These engineering strategies would eliminate non-human immunoglobulins from the formulation while preserving the rapid, non-covalent “plug-and-play” Fc-capture mechanism.

The in vitro results confirmed the essential functional benefits of this system. The ability of the UPs to specifically bind to soluble GFP, along with the observation of increased cellular uptake in Panc02 cells when functionalized with anti-Muc1/Muc4 antibodies, validates the core idea of targeted recognition. This targeted cellular interaction is especially important when considering highly expressed markers, such as Muc1 and Muc4, on pancreatic cancer cells. The enhanced uptake suggests that the UPs are capable of interacting with target cells in vitro, a necessary prerequisite for further therapeutic delivery investigation.

Despite the typical susceptibility of non-covalent adapters to displacement by serum proteins, the UP platform demonstrated robust stability by maintaining antigen-binding activity with no significant loss after 48 h of incubation in 10% FBS. Driven by the high thermodynamic affinity of the secondary-to-primary antibody interface, this resilient, Fc-mediated linkage preserves outward-facing Fab domains, supporting further investigation of the platform for in vivo targeted drug delivery applications.

Beyond in vitro resistance to serum proteins, the platform’s functional stability against competitive displacement by endogenous circulating IgG (Fig. S1H) is underpinned in vivo by high thermodynamic affinity and slow dissociation kinetics (k_off_). Moreover, displaying ~26 secondary antibodies per liposome creates high local avidity. Because the binding moieties are physically tethered to the same nanoscale lipid bilayer, transiently dissociated primary antibodies are restricted to a nanometer-scale reaction volume rather than escaping into the bulk circulation, substantially elevating their effective local concentration and driving rapid rebinding before systemic exchange can occur [43,44]. This kinetic retention preserves functional Fab presentation during first-pass transit and target-cell engagement.

The in vivo biodistribution data further demonstrate the platform’s versatility and specificity. The selective, statistically significant accumulation of anti-CD31 UPs in the lungs, without affecting clearance in the liver and spleen, provides strong proof of concept for targeted endothelial cell delivery. CD31 (PECAM-1) is a well-known marker for vascular endothelial cells [45]. Its high expression in the lungs supports the targeting observed.

It is important to note that the high absolute radiance observed in clearance organs such as the liver and kidneys is expected due to their central role in nanoparticle filtration and clearance. Consequently, absolute organ fluorescence does not necessarily reflect targeting specificity. Targeted UPs exhibited a statistically significant increase in target-site accumulation (lungs or tumors) compared with non-targeted controls (*p* < 0.0001), demonstrating that the surface-bound antibodies remained functional and accessible for target recognition.

While the enhanced pulmonary accumulation of αCD31-UPs demonstrates effective vascular engagement in the lung capillary bed, we acknowledge that αGFP-UPs served as the non-binding control in this study. Notably, primary clearance organs (liver and spleen) displayed comparable accumulation profiles across both targeted and control groups without evidence of RES divergence, indicating that non-specific Fc-mediated systemic clearance did not drive the observed pulmonary tropism. Nevertheless, future platform evaluations will incorporate matched non-targeting rat isotypes and in vivo receptor-blocking assays to dissect fine binding kinetics in the pulmonary microvasculature.

In the subcutaneous Panc02 model, dual αMuc1+4-UPs demonstrated a significant, ~3-fold increase in tumor fluorescence compared to non-targeted controls, while major clearance organs exhibited comparable accumulation between cohorts. This accumulation aligns with our in vitro findings, where αMuc1+4 decoration directly mediated cellular binding and internalization in high-mucin-expressing Panc02 cells. While passive accumulation through the EPR effect plays a role in tumor localization, these data indicate that active targeting provided by the UPs results in associated with enhanced accumulation profile compared to passive mechanisms. The ability to increase nanocarrier accumulation at the target site is important for the further development of targeted therapeutic delivery. To fully interpret this biodistribution profile, the statistically significant increase in cardiopulmonary accumulation observed for the dual-targeted αMuc1+4-UPs represents a recognized physical phenomenon in targeted nanocarriers. As described by Maruyama, Huang, and colleagues, high-density antibody decoration may promote transient micro-aggregation and slow down transit through pulmonary capillaries, potentially leading to temporary cardiopulmonary retention during first-pass circulation, while remaining compatible with tumor accumulation at the primary tumor target [46]. However, it is critical to acknowledge that enhanced tumor accumulation does not always correlate with improved clinical efficacy. As demonstrated in a comprehensive meta-analysis by Petersen et al., many liposomal formulations that show superior survival benefits in preclinical murine models fail to show a significant advantage in randomized clinical trials [47]. This translational gap is often attributed to the overestimation of the EPR effect in rapidly growing murine tumors compared to the more heterogeneous tumor microenvironment in patients. Furthermore, carrier-mediated immune modulation - such as the polarization of tumor-associated macrophages (TAMs) toward an immunosuppressive M2 phenotype - can inadvertently support therapeutic resistance, highlighting the need for rigorous immunological assessment in future platform iterations [47]. While dual targeting was chosen to account for mucin expression heterogeneity in PDAC, we acknowledge that single-targeted comparators (αMuc1 or αMuc4 alone) and in vivo receptor-blocking studies were not evaluated in this preliminary proof-of-concept. Future therapeutic payload efficacy studies will systematically delineate the relative contributions of single versus dual target engagement and validate receptor-specific blockade in vivo.

## 5 Conclusion

This study demonstrates the development and characterization of UPs as a versatile platform for the rapid and simple antibody-functionalization of liposomal surfaces. Through Fc-mediated binding, the UP platform bypasses the complexities of conventional chemical conjugation, promoting optimal antibody orientation and enhanced antigen recognition. In vitro experiments confirmed the ability of UPs to specifically bind target antigens and improve cellular uptake in Panc02 cells. Furthermore, in vivo biodistribution studies validated the platform’s capacity for tissue-specific accumulation, with selective targeting observed in both the pulmonary vasculature and murine pancreatic tumors.

Despite these promising results, it is important to acknowledge that this study serves as a preliminary proof-of-concept focused primarily on targeting and biodistribution. Although active targeting improves accumulation over the passive EPR effect, increased target-site accumulation alone does not guarantee therapeutic success, particularly given the complexity of the human tumor microenvironment. A current limitation of the platform is the absence of data demonstrating actual drug payload delivery or therapeutic outcomes. While the specific accumulation at target sites is a necessary condition for effective therapy, it does not address critical factors such as intracellular drug release, endosomal escape, or the physiological impact of a therapeutic agent. Future studies will therefore focus on incorporating therapeutic payloads, including chemotherapeutic agents or nucleic acids, into the UP core and evaluating their delivery, therapeutic efficacy, and safety profile in relevant disease models. Such studies will be essential to determine the translational potential of this platform for targeted drug delivery applications.

Taken together, the Fc-mediated immunofunctionalization strategy offers a powerful and flexible architecture for targeted nanomedicine. By streamlining the traditionally complex process of developing immunoliposomes, the UP platform provides a modular screening framework for the evaluation of targeted nanocarrier candidate designs against a wide range of pathological markers.

## Supplementary Material

**Appendix A. Supplementary data**

Supplementary data to this article can be found online at https://doi.org/10.1016/j.jconrel.2026.115319.

Supplementary

## Data Availability

The datasets and characterization files generated during and/or analyzed during the current study are deposited in the Zenodo repository under DOI: https://doi.org/10.5281/zenodo.22049308.
