# Supplementary material for: Rapid Fc-mediated surface modification of liposomes with antibodies for selective targeting of pancreatic tumors
Source: J Control Release. Author manuscript; Available in PMC 2026 Oct 11. (PMC7619544; doi:10.1016/j.jconrel.2026.115319)
Supplement: Supplementary file 1 [file EMS219691-supplement-Supplementary.docx]

**Supporting Information**

**Rapid Fc-Mediated Surface Modification of Liposomes with Antibodies for Selective Targeting of Pancreatic Tumors**

*Ibrahim Knani ^a, b, c*^,* *Yasmin Habib ^a, d^, Galoz Kaneti ^a^, Orly Shayderman ^a^, Mohammed Alyan ^a^, Tasneem Abu-Raiya ^a^, Jeny Shklover ^a^, Avi Schroeder ^a*^*

^a^ The Louis Family Laboratory for Targeted Drug Delivery and Personalized Medicine Technologies, Department of Chemical Engineering, Technion─Israel Institute of Technology, Haifa 3200003, Israel.

^b^ The Institute of Applied Research, The Galilee Society, Shefa-Amr, Israel.

^c^ Medical Research Institute, The Holy Family Hospital, Nazareth 1641100, affiliated with Azrieli Faculty of Medicine, Bar Ilan University, Safed 1311502, Israel.

^d^ The Norman Seiden Multidisciplinary Program for Nanoscience and Nanotechnology, Technion – Israel Institute of Technology, Haifa 32000, Israel

* **Corresponding authors**. avids@technion.ac.il (A. Schroeder), ibknani@gmail.com (I. Knani).

**Figure S1. A,** Representative autocorrelation decay curve, $g_{2}\left( \tau\right)-1$, as a function of time $\mathrm{lag}\left( \tau\right)$ for liposomal formulation in PBS. The smooth exponential fit demonstrates a high-quality DLS measurement, indicating a narrow size distribution. B, Tryptophan Emission Spectrum - antibody detection: The tryptophan emission spectrum peak at 340-350 nm is detected in the presence of liposomes, showing antibody binding to the liposome after Tangential Flow Filtration (TFF). C, Tryptophan quantification standard curve demonstrates a robust linear relationship (R^2^ = 0.9992) described by the equation y = 4588.2x + 908.44. D, Unbound antibody was efficiently removed from the sample via TFF. The purification efficacy was confirmed by measuring the tryptophan emission peak at 350 nm in the permeate fraction, indicating effective removal of free antibody. In contrast, the antibody exhibited stable binding to the liposomes and remained efficiently retained after the wash process (E). F, GFP emission spectrum: The GFP emission spectrum peaks at around 507 nm. This sensitivity makes fluorescence a valuable tool for detecting ligands or antigens. G, Unbound GFP was efficiently removed from the sample via Tangential Flow Filtration (TFF) using a hollow fiber membrane cassette with a specified membrane pore size of 50nm cut-off. The sample was subjected to a specified number of diafiltration cycles against a wash buffer of specified Buffer, PBS, to ensure high purity. H, Serum stability and functional antigen-binding retention of UPs. *p < 0.0001 relative to the corresponding controls.

**Muc4**

**Muc1**

**Figure S2.** Expression Analysis, Prognostic Value, and Tissue Distribution of Muc1 and Muc4. This figure presents a multi-faceted analysis of Muc1 and Muc4, along with the expression overview of PECAM1 (CD31), across various cancer types and normal/pre-cancerous tissues, utilizing public cancer genomics databases. **A and B, Kaplan-Meier Survival Curve for Muc1 and Muc4:** suggests that high Muc1 and Muc4 expressions are associated with significantly poorer prognosis. The number of patients at risk over time is indicated below the plot. **Data was obtained from the Kaplan-Meier database. C and D, Box plot displaying mRNA expression levels—quantified as Transcripts Per Million (TPM) of the *Muc1* and *Muc4* gene across a panel of healthy normal tissues (left, lighter boxes) and their corresponding primary tumors (right, darker/outlined boxes). Muc1 and Muc4 expression are markedly upregulated in pancreatic primary tumors compared to normal pancreatic tissue. Note: X-axis labels indicate specific tissues and tumor subtypes; red labels with an asterisk (*) denote statistically significant differences in expression between normal and tumor cohorts. Data was generated using the TNMplot platform (Bartha & Győrffy, 2026,** [**https://tnmplot.com/analysis/**](https://tnmplot.com/analysis/)**). E, PECAM1 (CD31) Protein Expression Overview:** Bar chart illustrating the relative protein expression levels of PECAM1 (CD31), a marker for endothelial cells, across a wide range of human organs. The expression levels (Not detected, Low, Medium, High) confirm their dominant presence in the lungs. Data was sourced from the Human Protein Atlas (HPA, <https://www.proteinatlas.org/>).

**Figure S3**. In Vivo Fluorescence Imaging of Organ Biodistribution of targeted αCD31-UPs. **A,** In vivo fluorescence image of excised lungs from untreated (Cont), non-targeted Liposome (Lipo), and CD31-targeted UPs groups (αCD31-UPs) following i.v. administration of 200 µL in C57BL/6 mice. Lipo and αCD31-UPs groups were both labeled with Cy5. The images were obtained at 4 hours post-injection by the MILabs VECTor7 system, utilizing filter settings (Cy5: λ_ex: 625; λ_em: 661 nm). Fluorescence intensity is represented by a pseudocolor scale (blue to red, indicating low to high signal). The heat map indicates fluorescence intensity in arbitrary digital units (ADU). **B,** in vivo fluorescence image of excised lungs and kidneys from Cont, Lipo, and αCD31-UPs groups. **C and D, in Vivo Images of lungs and kidneys (Replicate): another independent biological repeat demonstrating consistent baseline fluorescence for the Lipo and** αCD31-UPs groups**.**
